# Transcriptomic identification of genes expressed in invasive *S. aureus* diabetic foot ulcer infection

**DOI:** 10.3389/fcimb.2023.1198115

**Published:** 2023-06-26

**Authors:** Taiwo Samuel Agidigbi, Hyuk-Kwon Kwon, James R. Knight, Dejian Zhao, Francis Y. Lee, Irvin Oh

**Affiliations:** ^1^ Department of Orthopedics and Rehabilitation, Yale School of Medicine, New Haven, CT, United States; ^2^ Division of Life Science, Gyeongsang National University, Jinju, Republic of Korea; ^3^ Yale Center for Genome Analysis, Department of Genetics, Yale School of Medicine, New Haven, CT, United States

**Keywords:** DFU, *S. aureus*-infection, transcriptome, PBMC, wound healing, host-immunity

## Abstract

**Introduction:**

Infection in diabetic foot ulcers (DFUs) is one of the major complications associated with patients with diabetes. *Staphylococcus aureus* is the most common offending pathogen in patients with infected DFU. Previous studies have suggested the application of species-specific antibodies against *S. aureus* for diagnosis and monitoring treatment response. Early and accurate identification of the main pathogen is critical for management of DFU infection. Understanding the host immune response against species-specific infection may facilitate diagnosis and may suggest potential intervention options to promote healing infected DFUs. We sought to investigate evolving host transcriptome associated with surgical treatment of *S. aureus*– infected DFU.

**Methods:**

This study compared the transcriptome profile of 21 patients with *S. aureus*– infected DFU who underwent initial foot salvage therapy with irrigation and debridement followed by intravenous antibiotic therapy. Blood samples were collected at the recruitment (0 weeks) and 8 weeks after therapy to isolate peripheral blood mononuclear cells (PBMCs). We analyzed the PBMC expression of transcriptomes at two different time points (0 versus 8 weeks). Subjects were further divided into two groups at 8 weeks: healed (n = 17, 80.95%) versus non-healed (n = 4, 19.05%) based on the wound healing status. DESeq2 differential gene analysis was performed.

**Results and discussion:**

An increased expression of *IGHG1*, *IGHG2*, *IGHG3*, *IGLV3-21*, and *IGLV6-57* was noted during active infection at 0 weeks compared with that at 8 weeks. Lysine- and arginine-rich histones (*HIST1H2AJ*, *HIST1H2AL*, *HIST1H2BM*, *HIST1H3B*, and *HIST1H3G*) were upregulated at the initial phase of active infection at 0 weeks. *CD177* and *RRM2* were also upregulated at the initial phase of active infection (0 weeks) compared with that at 8 weeks of follow-up. Genes of heat shock protein members (*HSPA1A*, *HSPE1*, and *HSP90B1*) were high in not healed patients compared with that in healed patients 8 weeks after therapy. The outcome of our study suggests that the identification of genes evolution based on a transcriptomic profiling could be a useful tool for diagnosing infection and assessing severity and host immune response to therapies.

## 1 Introduction

Infection of diabetic foot ulcer (DFU) presents a significant social and economic burden to our society. Diabetes and its associated infection affect an estimated 9.3% of people in the United States, accounting for more than 20% of all hospitalization (Kim and Boye, 2009; Skrepnek et al., 2017; Schmidt, 2022; McDermott et al., 2023). They frequently result in prolonged hospitalization, poor clinical outcomes, readmission, and, in extreme cases, amputation (Herber et al., 2007; Phillips et al., 2016; Armstrong et al., 2017; Skrepnek et al., 2017; Smith-Strom et al., 2017; Polikandrioti et al., 2020; Raffetto et al., 2020; Zhao et al., 2020; Tai et al., 2021; Guo et al., 2023). Infection or re-current infection of DFU can have an impact on quality of life and present social challenges and a declined in psychological wellbeing (Phillips et al., 2016; Egan et al., 2020; Guo et al., 2023). *Staphylococcus aureus* is the most common pathogen (70%–80%) found in patients with DFU infection (Macdonald et al., 2020). The main challenge in treating musculoskeletal infection (MSKI) is the identification of main pathogen (Lipsky et al., 2012; Heravi et al., 2020; Lipof et al., 2021). False-negative or false-positive cultures in polymicrobial MSKI may result in inappropriate antibiotic coverage plan, subsequent recurrent infection, and loss of the limb. Although inflammatory markers, such as white blood cell count (WBC), erythrocyte sedimentation rate (ESR), and C-reactive protein (CRP), have been utilized to diagnose and monitor treatment response, they are non-specific (Masters et al., 2019; Grondman et al., 2020; Lipof et al., 2021).

Previous investigations have demonstrated an increased host production of *S. aureus–* specific antibodies in patients with DFU. Specifically, antibodies against iron- regulated surface determinant (IsdB and IsdH) protein, recombinant staphylococcal complement inhibitor, and the recombinant clumping factors (ClfA and ClfB) were suggested as biomarker candidates to diagnose and monitor treatment response (Masters et al., 2019; Heravi et al., 2020; Choi et al., 2021; Hao et al., 2021; Lipof et al., 2021; Wang et al., 2021; Fritz et al., 2022). These endeavors to characterize humoral responses to *S. aureus* infection with the use of immunoassay techniques have shown some potential for clinical application, but there are certain limitations (Masters et al., 2019; Choi et al., 2021; Hao et al., 2021; Fritz et al., 2022). Because of the historical abundance of these antibodies produced by host’s previous exposure to infection, the elevated serum titers may not accurately reflect the state of the current infection.

Advancements in bioinformatics have suggested using RNA- seq technology to accelerate the knowledge on transcriptomic analysis. By profiling the transcriptome landscape of cells in heterogeneous tissues, it provides a deep insight into cell function and disease pathophysiology (Jiang et al., 2015). Transcriptome research focuses on mRNA and non-coding RNA acting as a regulator to numerous proteins and gene function/structure and reveals specific molecular mechanisms of disease occurrence (Jiang et al., 2015; Stubbington et al., 2017). The new technology paradigm provides a greater specificity for studying the immune response. The shift from the use of convectional culture-dependent methodologies and serum antibody levels to transcriptome analysis may help us better understand DFU infection, patient outcomes, and wound progression. Few research studies have recently elucidated the role of RNA-seq/transcriptomes in DFU (Theocharidis et al., 2020; Wang et al., 2020; Li Y, et al., 2022; Theocharidis et al., 2022; Sandoval-Schaefer et al., 2023). Transcriptome data analysis, for example, has been utilized to identify gene expression profiles, biological signal pathways, and biomarkers in various diseases such as cancer, tuberculosis, neuronal dysfunction, autoimmune diseases, and COVID-19 prognosis (Baine et al., 2011; Islam et al., 2017; Bellaver et al., 2019; Wang et al., 2019; Xiong et al., 2020; Zheng et al., 2020; Fang et al., 2021; Han et al., 2021; Ascoli et al., 2022; Ghosh et al., 2022; Maleknia et al., 2022; Mendelsohn et al., 2022; Zimmer et al., 2022). Theocharidis et al. recently reported the use of RNA-seq RNA-Seq to elucidate gene expression associated with wound healing in the single-cell transcriptome landscape of DFU in human and mice. The above study focuses on wound healing in DFU. However, in our study, we sought to investigate whether the transcriptome could be used to diagnose infected DFU. Our objective is to develop a more accurate transcriptome-based test specific for infection and its application for diagnosis and prognostication potential. Probing how host immune system responds to infection in DFU through the variability in gene expression will increase our knowledge in diagnosis and aid clinical decision in management of MSKI.

We aimed to analyze evolving human peripheral blood mononuclear cell (PBMC) transcriptome associated with treatment of clinically infected DFU to identify the genes involved in immunologic response to invasive *S. aureus* DFU infection. We sought to identify up- and downregulated genes among healed patients at the time of active infection (0 weeks) vs. 8 weeks after initiation of therapy. Secondary outcome was to investigate whether certain gene expression (singly or in conjunction with others) could serve as a prognostic indicator for resolution of infection and eventual wound healing.

## 2 Materials and methods

### 2.1 Ethics statement

This study was approved by the Yale Human Research Protection and Ethics Committee (Reference No. 2000030908). Informed consents were obtained from all patients enrolled.

### 2.2 Patient population and antibiotics regimen

Twenty-one patients with infected DFU whose bone culture obtained from the base of infected wound showed a growth of *S. aureus* formed the basis of the study. At the start of therapy, blood samples were taken to measure hemoglobin A1C, WBC, CRP, and ESR. All patients underwent initial foot salvage therapy with irrigation and debridement. Prior to surgery, intravenous (IV) antibiotics were withheld for all patients until intraoperative specimens were obtained for culture. Once obtained, a broad-spectrum IV antibiotic, such as piperacillin/tazobactam (Zosyn), was administered and continued until the culture results were available or until the regimen was changed by a consulting infectious disease (ID) specialist. For patients with a history of methicillin-resistant *S. aureus* infection, vancomycin was initiated instead. Histologic evaluation of resection margin was performed for all patients who underwent amputation or bony resection procedure. If a clear margin was confirmed, then patients were kept on oral antibiotics for additional 2 weeks after surgery to eliminate any residual infection in the soft tissue. However, if the resection margin was determined to be contaminated, then a culture-based targeted long-term (6 weeks) IV or oral antibiotics was administered as determined by the ID specialist. DFU infection and wound healing status were classified as healed vs. not healed based on wound status (size), clinical sign, and symptoms of infection (Wagner, 1981).

### 2.3 Sample collection

During wound debridement procedure, two pea-sized grossly infected bone samples were aseptically collected at the base of DFU (Oh et al., 2018; Choi et al., 2021). The samples were then separated into two sterile vials: One was transferred to the microbiology laboratory for standard culture, and the other vial was immediately stored in a −80°C. The DNA was extracted, and a quantitative polymerase chain reaction (qPCR) was performed with specific primers to determine the bacterial burden and antibiotic resistance genes.

### 2.4 Microbiological analysis to identify bacterial species

A method for microbial identification previously described (Harmsen et al., 2003; Oh et al., 2018; Choi et al., 2021) was adopted. Briefly, we did microbial culture as a standard practice and bacterial abundance was determined by real-time qPCR using bacterial 16S primers, probe, and cloned plasmid standards. The region of bacterial 16S rRNA from V1 to V3 was amplified from extracted DNA sample via dual-indexed coded primers and Phusion high-fidelity polymerase (Thermo Fisher, Inc.). With the use of SequalPrep normalization plates (Life Technologies), all PCR amplicons were purified, normalized, pooled, and validated on an Agilent bioanalyzer (2200 TapeStation, D1000 tape or HSD1000 tape). The final library was paired end and sequenced on an Illumina MiSeq (2 × 300 bp). In each amplification and sequencing run, a blank sample was run through the extraction, amplification, and sequencing steps as a negative control, whereas positive controls included bacterial genomic DNA from a variety of genera. We identified different bacteria species from the polymicrobial infection of the DFU such as *S. aureus*, *Enterococcus faecalis*, *Proteus mirabilis*, *Streptococcus agalactiae*, and *Serratia marcescens* from different patient’s samples. Because *S. aureus* alone accounted for about 70% of MSKI, we are unsure that only samples that were *S. aureus* positive were included in this research. To further confirm that *S. aureus* is present in all the selected infected samples, we genotyped bacterial cultures for the staphylococcal virulence gene *spa*, infected samples were classified on the basis of the outcome of the result as PCR ^+^ or PCR^−^ for *S. aureus*.

### 2.5 PBMC isolation

Blood samples from patients with DFU infection were collected at the enrollment (0 weeks) and at 8 weeks after initiation of treatment. PBMCs were isolated by density gradient centrifugation. Blood was gentle mixed by inversion several times to ensure a homogeneous suspension. The whole blood was mixed with equal volume of Phosphate-buffered saline (PBS) in ratio 1:1, gently layered the mixed blood-PBS on top of the density gradient medium (lyphoprep), and centrifuged at 1,500 *g* for 25 min at room temperature. PBMCs were harvest by inserting the pipette directly through the upper plasma layer to the mononuclear cells at the interface. The PBMC aliquot was stored in at −80°C for further analysis.

### 2.6 RNA-seq and differential gene expression analysis of the PBMCs

Methods for RNA-seq and differential gene expression analysis described were adopted with slight modification (Pertea et al., 2015). Briefly, gene expression levels were quantified using StringTie v1.3.3b with gene models (v27) from the GENCODE project. PBMCs were lysed on ice for 5 min in 10 mM Tris-HCl (pH 7.5), 10 mM NaCl, 0.2 mM Ethylene-diamine-tetra acetic acid (EDTA), and 0.05% NP-40, and nuclei were spun at 2,500*g* for 3 min before being resuspended in QIAzol for RNA isolation using a miRNeasy kit, as directed by the manufacturer (Qiagen). The RNA-seq library was constructed using the Illumina TruSeq RNA Sample Preparation Kit v2 and sequenced on one lane of the HiSeq 2000 platform (100 bp, paired-end). Low- quality reads were trimmed, and adaptor contamination was removed using Trim Galore (v0.5.0). Trimmed reads were mapped to the human reference genome (hg38) using HISAT2 v2.1.0 and differentially expressed genes were identified using DESeq2 v 1.22.1 (Love et al., 2014; Pertea et al., 2015; Kim et al., 2019).

### 2.7 Statistical and ingenuity pathway analysis

Human PBMC Differentially Expressed (DE) gene lists were filtered using an Log_2_ Fold Change (LFC) cutoff to measure the statistical dependence between two variables (0 and 8 weeks) for which a value of ≥ 1 indicates a positive correlation and ≤ −1 indicates a negative correlation. Positively and negatively correlated genes that were statistically significant with reference to ≤ 0.05 False Discovery Rate (FDR) cut off value were uploaded into Ingenuity Pathway Analysis (IPA) (Qiagen), (https://digitalinsights.qiagen.com/products-overview/discovery-insights-portfolio/analysis-andvisualization/qiagen-ipa/) (Kramer et al., 2014). IPA software used Fisher’s exact binomial confidence interval test to detect statistically significant up- or downregulated genes for healed samples at 0 weeks before initiation of antibiotic vs. 8 weeks of follow-up after therapy and in healed vs. not healed at 8 weeks after antibiotic therapy.

### 2.8 Data availability

Transcriptomics of the RNA-seq data have been deposited in the NCBI’s Gene Expression Omnibus (GEO) and can be accessed using GEO accession number GSE230426.

## 3 Results

### 3.1 Correlation of clinical variables

**Figure 1:**
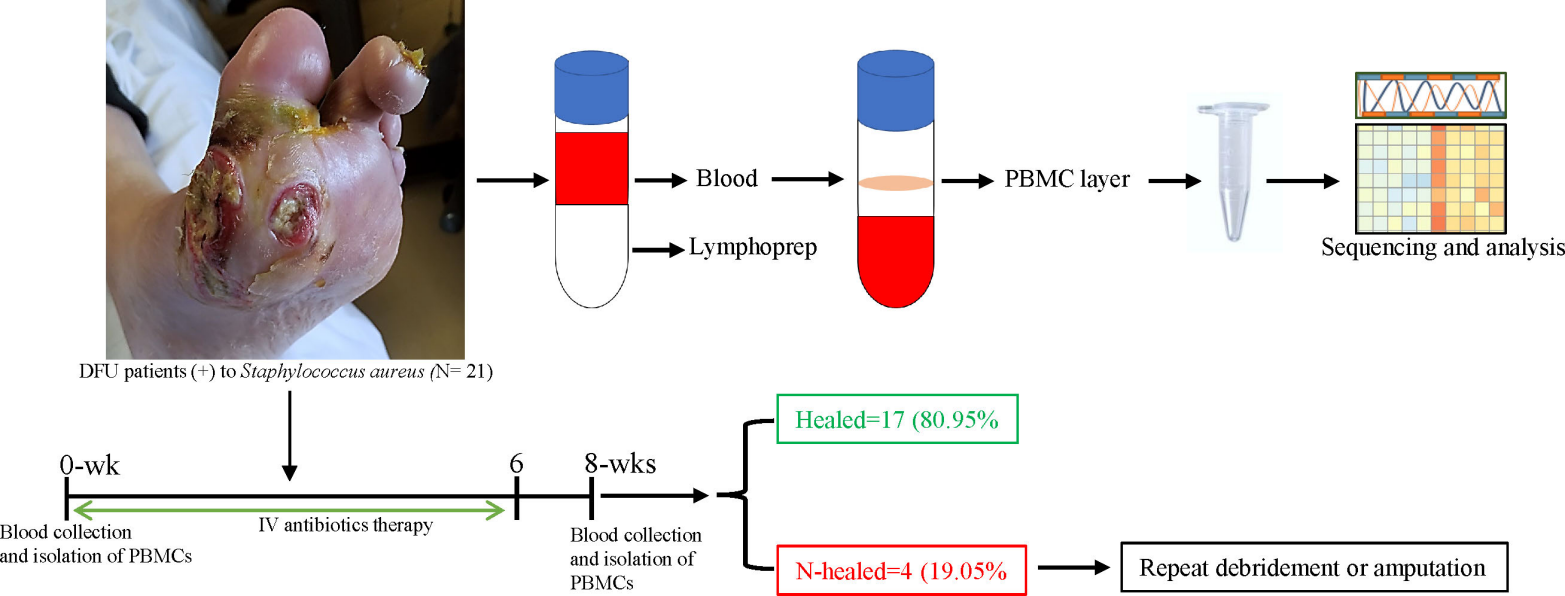
Schematic overview of the study design. Whole -blood sample was collected from patients with *S. aureus*– infected DFU before initiation of antibiotics (0 weeks) and after therapy (8 weeks) to isolate PBMC. Blood samples were mix with PBS (1:1), lymphoprep density gradients was used to separate PBMC at the interphase, and PBMC samples were harvested and used for RNA and transcriptome analysis. After 8 weeks of follow -up, subjects were divided into two groups: healed (n = 17) versus non-healed (n = 3) based on infection symptoms and the wound healing status; non-healers were either debrided again or undergo amputation.

We analyzed 42 samples from 21 (n = 21) patients with *S. aureus*– infected DFU who underwent surgical debridement/treatment. Infection status was clinically determined on the basis of the assessment of 12 signs and symptoms (Gardner et al., 2013). Wound healing status (size), presence of infection such as purulent exudate, erythema, pain, or offensive odor was documented at 0 and 8 weeks after initiation of therapy. In this study, n = 17 (80.95%) patients clinically healed the infection at 8 weeks of follow-up, whereas n = 4 (19.05%) patients failed to heal. Those patients who failed to heal underwent repeat debridement or amputation (**Figure 1**). Analysis of our results was based on the comparison between 0 and 8 weeks among patients that were healed (n = 17) and between healers (n = 17) and non-healers (n = 3) at 8 weeks after antibiotic therapy. One sample (n = 1) was contaminated and was excluded from further analysis.

### 3.2 Immunoglobulin heavy G chain upregulated during active infection

**Figure 2:**
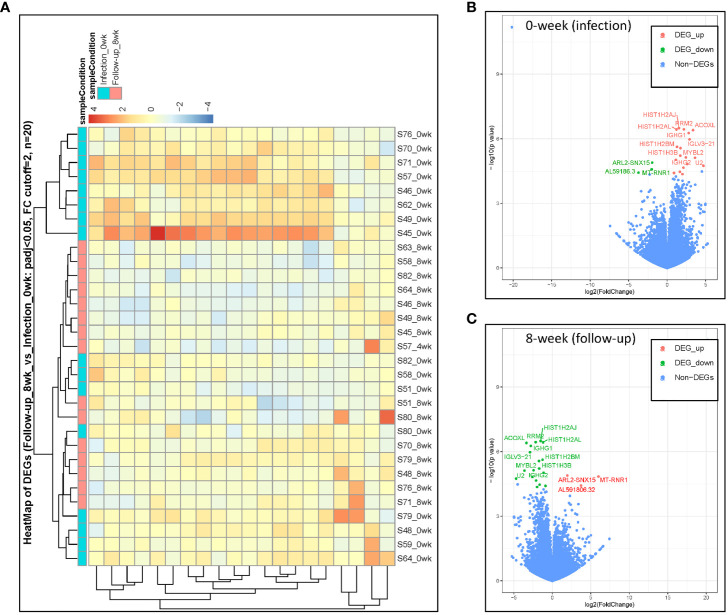
DESeq2 analysis was used to characterize the transcriptomes of host PBMC mRNA gene expression among healed patients (n = 17). **(A)** Heatmap identification of gene pattern of expression. **(B, C)** DE volcano plot of gene expression at 0 weeks during active infection compared with that at 8 weeks of follow-up among healed patients. Transcripts of selected upregulated genes colored in red, whereas downregulated genes are colored in green.

**Figure 3:**
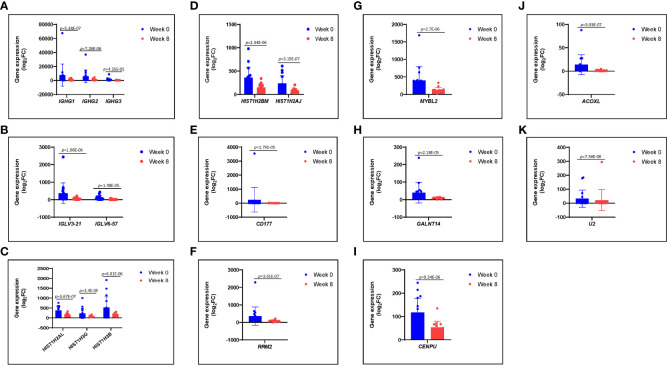
**(A–K)** Differences in gene expression patterns among healed patients (n = 17) during active infection at 0 (blue) and 8 (red) weeks. These genes were significantly expressed during active infection, but they were downregulated at 8 weeks following antibiotic therapy.

Immunoglobulins are glycoproteins that are exclusively produced by activated B lymphocytes and plasma cells and mediate the humoral response to pathogens. We identified differences in PBMC mRNA gene expression/pattern as demonstrated in the RNA sequence heatmap and volcano plot (**Figure 2**). The analysis showed a significant variation in the transcriptome profile observed among the members of immunoglobulin gamma heavy chain (*IGHG1*, *IGHG2*, and *IGHG3*). Among the groups that were healed of the infection after antibiotic therapy (n = 17), we observed a higher expression of *IGHG1*, *IGHG2*, and *IGHG3* in the active phase of infection at 0 week, and the gene expression of these immunoglobulins was downregulated at 8 weeks of follow-up (p = 5.38 × 10^−7^, 7.26 × 10^−6^, and 4.35 × 10^−5^ with corresponding FDR values of 0.002183, 0.013948, and 0.042061, respectively). These findings indicate a significant association between evolving expression of these immunoglobulins and resolution of infection (**Figure 3A**; **Table 1**).

**Table 1:** Top differentially expressed genes at 8 weeks of follow-up vs. 0 weeks.

Genes expression	0 weeks (infection)	8 weeks (treatment)	−Log2FC	p-value	FDR
*CENPU*	↑	↓	1.136585029	9.43E-06	0.01594
*HIST1H2AL*	↑	↓	1.198035266	3.67E-07	0.00199
*HIST1H2BM*	↑	↓	1.274861243	2.34E-06	0.00679
*HIST1H2AJ*	↑	↓	1.524669693	3.19E-07	0.00199
*HIST1H3G*	↑	↓	1.655786672	3.4E-05	0.03831
*HIST1H3B*	↑	↓	1.706188302	6.01E-06	0.01355
*MYBL2*	↑	↓	1.732445906	2.7E-06	0.00684
*IGHG3*	↑	↓	1.98479736	4.35E-05	0.04206
*GALNT14*	↑	↓	2.11040763	2.18E-05	0.02765
*RRM2*	↑	↓	2.168273585	3.61E-07	0.00199
*IGHG2*	↑	↓	2.432628837	7.26E-06	0.01395
*IGLV6-57*	↑	↓	2.599064812	1.48E-05	0.02142
*IGHG1*	↑	↓	2.796831616	5.38E-07	0.00218
*IGLV3-21*	↑	↓	2.907367508	1.06E-06	0.00357
*ACOXL*	↑	↓	3.366341758	3.93E-07	0.00199
*U2*	↑	↓	3.618387229	7.56E-06	0.01395
*CD177*	↑	↓	4.710844858	1.79E-05	0.02415
*MT-RNR1*	↓	↑	1.94448761	1.27E-05	0.01982
*AL591806.3*	↓	↑	2.00620946	2.61E-05	0.03120
*ARL2-SNX15*	↓	↑	3.72998307	3.72E-05	0.03917

### 3.3 *IGLV3*-21 and *IGLV6*-57 downregulated after antibiotic therapy

Recently, studies have found significant variation in immunoglobulin loci, as well as links between germline immunoglobulin families and humoral response through B- cell receptors and antibodies expressed by B cells (Pennell et al., 2023). We found a significant increase in expression of *IGLV3-21* and *IGLV6-57* genes during active *S. aureus* DFU infection at 0 weeks; these genes and other immunoglobulin families of *IGHG* remain higher as observed among the patients (n = 3) with unresolved infection (data not shown) but became downregulated at 8 weeks associated with resolution of infection in healed patients (n = 17) after antibiotic therapy (p = 1.06E-06 and 1.48-E05) with corresponding FDR values of 0.003573 and 0.02142, respectively (**Figure 3B**; **Table 1**).

### 3.4 Core histone genes downregulated after antibiotic therapy

Histones are classified as lysine-rich (H1, H2A, and H2B) or arginine-rich (H3 and H4), and they play a significant role in DNA condensation (Li X, et al., 2022). They accumulate in the cytoplasm and on the plasma membrane, where they perform other functions such as antimicrobial activity. Quantitative transcriptome analyses with mRNA samples isolated from the PBMCs revealed that core histone genes such as *HIST1H2AJ*, *HIST1H2AL*, *HIST1H2BM*, *HIST1H3B*, and *HIST1H3G* were upregulated during active infection at 0 weeks and downregulated at 8 weeks following antibiotic therapy. Among the patients that were healed (n = 17), our data identified statistically significant downregulation of the aforementioned histone genes at 8 weeks (p = 3.19E-07, 3.67E-07, 2.34E-06, 6.01E-06, and 3.4E-05 with FDR values of 0.001994, 0.001994, 0.006794, 0.01354, and 0.038314, respectively) when compared with the active infection at 0 weeks (**Figures 3C, D**; **Table 1**). Despite the limitation of small sample size and variation in the severity of illness among the three unhealed patients compared with the healed patients, expression of histone genes remained relatively high in unresolved infection after 8 weeks (data not shown).

### 3.5 Cluster of differentiation 177 upregulated during active infection at 0 weeks

*CD177* is a well-known neutrophil antigen/cell surface glycoprotein involved in neutrophil activation and migration. In response to inflammatory stimuli, neutrophils migrate from the circulating blood to infected tissues, where they protect the host by phagocytosing, killing, and digesting bacterial and fungal pathogens. In the healed group (n = 17), *CD177* was highly expressed in response to active infection at 0 weeks and became downregulated at 8 weeks following antibiotic therapy. *CD177* had the highest fold change among other genes at 8 weeks when compared with that at 0 weeks with −Log_2_FC of 4.7108 (p = 1.79E-05) with FDR value of 0.024148 (**Figure 3E**; **Table 1**).

### 3.6 *RRM2* and *MYBL2* upregulated during active infection at 0 weeks

Ribonucleotide reductase M2 (*RRM2*) is a subunit of ribonucleotide reductase, which is required for DNA replication and damage repair, whereas *MYBL2* is a transcription factor in the Myb-related protein B (MYB) family that regulates cell cycle progression, survival, and differentiation. Our results also identified *RRM2* and *MYBL2* as part of differentially expressed mRNAs gene associated with PBMCs from patients with infected DFU. In the healed group (n = 17), *RRM2* and *MYBL2* were significantly upregulated during active infection at 0 weeks but became downregulated at 8 weeks following therapy (p = 3.61E-07 and 2.7E-06 with corresponding FDR values of 0.001994 and 0.006835, respectively) (**Figures 3F, G**; **Table 1**). In addition, the expression of *RRM2* gene was also higher among the unhealed patients (n = 3) obtaining a significant p-value of 9.177E-05 with log_2_FC of 2.403 but failed to obtain a significant p-adjusted value that we taught might be due to small sample size and variation in infection severity among the unhealed patients when compared with healed patients (data not shown). The expression of *RRM2* was significantly upregulated in parallel with *CD177* and *MYBL2*. Other genes that were significantly upregulated during active infection include *GALNT14*, *CENPU*, *ACOXL*, and *U2* (**Figures 3H–K**).

### 3.7 Heat shock protein upregulated in unresolved infection after antibiotic therapy

We reported that, following antibiotic therapy, subjects were divided into two groups: healed (n = 17) and not healed (n = 3) at 8 weeks. We compared the PBMC mRNA gene transcripts to check the difference in gene expression between these two groups (healed vs. not healed). Among the notable genes that were differentially expressed, we observed a significant increase in the gene expression of heat shock protein (HSP) members such as *HSPA1A*, *HSPE1*, and *HSP90B1* in unhealed patients (n = 3) when compared with healed patients (n =1 7) at 8 weeks after antibiotic therapy (p = 3.284E-05, 2.188E-05, and 2.355E-05 with corresponding FDR values of 1.8444E-10, 3.401E-03, and 3.401E-03, respectively). Other differentially expressed genes that were either downregulated or upregulated in healed and not healed patients after therapy at 8 weeks are listed in **Table 2**.

**Table 2:** Top differentially expressed genes at 8 weeks of follow-up.

Genes expression	Not healed	Healed	−Log2FC	p-value	FDR
*HSPA1A*	↑	↓	7.647462434	1.024E-14	1.844E-10
*AC018755.2*	↑	↓	5.18984005	8.283E-08	2.983E-05
*TDRD9*	↑	↓	3.573234544	8.927E-06	1.786E-03
*IGKJ5*	↑	↓	3.508200073	3.284E-05	3.602E-03
*HSPG2*	↑	↓	3.227681846	4.339E-07	1.302E-04
*HK2*	↑	↓	3.06708647	2.097E-05	3.401E-03
*GPI*	↑	↓	2.428346225	4.820E-06	1.085E-03
*CPAMD8*	↑	↓	2.423884532	3.325E-05	3.602E-03
*WSB1*	↑	↓	2.366481623	3.613E-05	3.613E-03
*NCKIPSD*	↑	↓	2.017872607	3.122E-05	3.602E-03
*HSPE1*	↑	↓	1.990428389	2.188E-05	3.401E-03
*MCTP2*	↑	↓	1.858777058	3.401E-05	3.602E-03
*HSP90B1*	↑	↓	1.592076216	2.355E-05	3.401E-03
*AL031714.1*	↓	↑	2.305365574	2.456E-05	3.401E-03
*AC068205.3*	↓	↑	6.494810093	3.973E-05	3.764E-03
*C1QTNF3-AMACR*	↓	↑	7.485904955	1.067E-06	2.744E-04
*CAMK2A*	↓	↑	24.48659889	1.436E-09	6.464E-06
*ARSI*	↓	↑	25.65541914	1.055E-11	6.329E-08
*XIST*	↓	↑	30.00000000	1.225E-13	1.102E-09

## 4 Discussion

In this study, we performed RNA- seq transcriptome analysis of PBMCs to accurately and systematically profile gene expression in PBMCs of patients with *S. aureus*– infected DFU at the time of active infection (0 weeks) and after antibiotic therapy (8 weeks). Developing a robust system for earlier diagnosis of *S. aureus* infection is essential for diagnosis, treatment, and prognostication. Unlike the report by Theocharidis et al. and other authors on the genes profile of PBMC single-cell RNA that focused primarily on fibroblast- and keratinocyte-associated genes (wound healing) between patients with DFU whose ulcers heal (DFU healers) and those who do not heal (DFU non-healers), we focus primarily on profiling the genes that were differentially expressed in patients with infected DFU at different time points (0 vs. 8 weeks) to improve our understanding of earlier diagnosis, prognostication, and monitoring treatment response. We have identified various genes encoding different immunoglobulin and chemokines that are involved in the progression and healing of wounds in the PBMC samples of patients with *S. aureus*– infected DFU. Our findings suggest a complex interplay of host immune response via neutrophile and immunoglobulins.

We identified several members of histone genes that are highly regulated during *S. aureus*– infected DFU wound healing. Among the patients that were healed, we found that core histones such as *HIST1H2AJ*, *HIST1H2AL*, *HIST1H2BM*, *HIST1H3B*, and *HIST1H3G* were highly expressed during active infection at 0 weeks (and among the three unhealed patients) but became downregulated at 8 weeks after treatment follow-up, indicating that histone may play a significant role in host immune response to infection resolution. Histones are classified as lysine-rich (H1, H2A, and H2B) or arginine-rich (H3 and H4); among these two groups, H2A, H2B, H3, and H4 are known as “core histones”, whereas H1 is known as “linker histones” (Felsenfeld and Groudine, 2003; Li X, et al., 2022). Histones are the main components of nucleosomes, and they play a role in the packaging and arranging of DNA into functional units. However, there is growing evidence that histones can be found outside the nucleus and become extranuclear histones. These “extranuclear” histones have reported to enhance host defense functions and contribute to inflammatory responses (Urban et al., 2009; Venkatesh and Workman, 2015; Frydman et al., 2020).

Parallel to upregulation of core histones, the *CD177* gene (a neutrophil-specific marker), *MYBL2* and *RRM2* were highly expressed during the active phase of infection (0 weeks) and the expressed genes were reduced at 8 weeks after therapy. These data are consistent with reported positive correlation of *RRM2* with most chemokines and chemokine receptors (Zhou et al., 2022). Histones can act as antimicrobial peptides and directly kill bacteria and other pathogens in a variety of animal hosts. In addition, histones can trigger inflammatory responses, in some cases, acting through Toll-like receptors (Morita et al., 2013; Hoeksema et al., 2016). In addition, phagocytes are essential components of innate immune defense against infectious pathogens, and there is mounting evidence that histones and phagocytes have interactions (Li X, et al., 2022). Once phagocytosed, *S. aureus* is exposed to a variety of toxic products (NO, H_2_O_2_, superoxide and hydroxyl radicals, hydrolases, and proteolytic enzymes) that kill and degrade the engulfed bacteria.

Neutrophil extracellular traps (NETs) are fibrous structures with decondensed chromatin networks released from activated neutrophils during infection, which can capture and eliminate pathogens (Papayannopoulos, 2018; Song et al., 2019; Tan et al., 2021). NETs contain all core histones (H2A, H2B, H3, and H4), accounting for 65%–70% of all NET-related proteins; although their functions have not been fully elucidated, the presence of histones in NET structure suggested that histones could act as antimicrobial agents in NETs. Histones and their fragments play a vital role in host defense by binding to bacterial nucleic acid and lipopolysaccharide and by changing the permeability of bacterial cell membrane, and the antibacterial role of histones in different species is becoming more widely recognized (Howell et al., 2003; Patat et al., 2004; Lemaire et al., 2008; Urban et al., 2009; Mehta and Jeffrey, 2015; Shrestha et al., 2019; Doolin et al., 2020; Duong et al., 2020). High expression of *CD177* and histones at 0 weeks may suggest a strong association with the severity of *S. aureus* infection.

**Figure 4:**
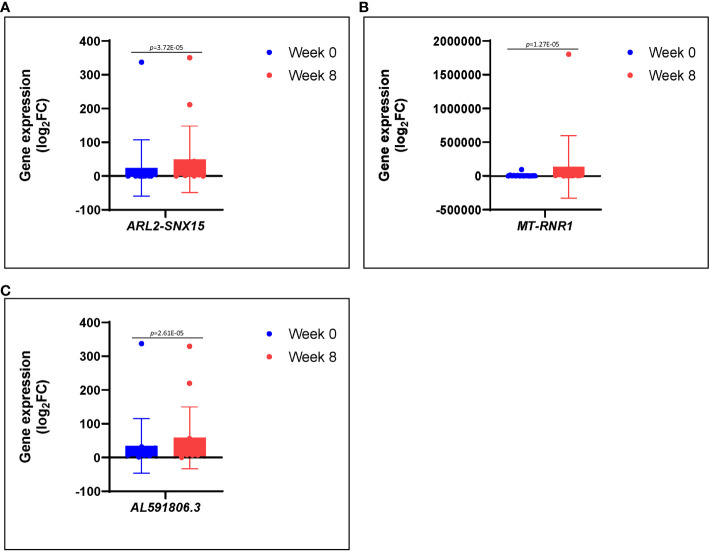
**(A–C)** Differences in gene expression patterns among healed patients (n = 17) during active infection at 0 (blue) and 8 (red) weeks. These genes were significantly repressed during active infection, but they were upregulated at 8 weeks following antibiotic therapy.

We noted a high level expression of *RRM2*, *MYBL2*, and some other genes such as *GALNT14*, *CENPU*, *ACOXL*, and *U2* at 0 weeks during active phase of infection, which were downregulated at 8 weeks after therapy (**Table 1**). We also observed a low level expression of *ARL2-SNX15*, *MT-RNR1*, and *AL591806*.3 at 0 weeks during active infection, which were upregulated at 8 weeks after therapy (**Figures 4A–C**; **Table 1**). The role of these genes in *S. aureus*– infected DFU wound healing and resolving infection is not completely understood. Among others, *RRM2* has been reported as a member of the ferric iron-binding ferritin superfamily and that *RRM2* engaged in the iron metabolism in hepatocellular carcinoma (Key et al., 2020; Shen et al., 2021). Although we did not know whether *RRM2* is important for *S. aureus* pathogenicity, we observed a high expression of this seemly important gene among the three unhealed patients. Given that iron (heme) acquisition is a pathognomonic finding of invasive *S. aureus* infection, it would be prudent to further investigate factors associated with iron metabolism, which may potentially involve *RRM2.*

We reported a high expression of IGHG family (*IGHG1*, *IGHG2*, and *IGHG3*) and IGLV family (*IGLV3*-21 and *IGLV6*-57) at 0 weeks, which are clinically correlate with active infection. Conversely, *IGHG* and *IGLV* expression was reduced at 8 weeks after therapy but remain higher in unresolved infection. Because neutrophil- associated histones and *CD117* were also reduced at 8 weeks, these findings reflect a reduction of *S. aureus* infection burden at 8 weeks after therapy. Our finding suggests that diverse *IGHG* and *IGLV* family may contribute to a protective host immune response against *S. aureus* infection in DFU and may serve as a potential biomarker for diagnosis and prognostication of infection and wound healing.

In addition, we noted a significant elevation in HSP levels in unresolved infection (not healed n = 3) compared with healed patients (n = 17) at 8 week after antibiotic therapy (**Table 2**). The presence of high levels of inducible HSPs in unhealed patients may be due to the continuous release of virulence machineries by *S. aureus* and the host immune response to unresolved infection. In line with our observation, HSPs have been reported in cellular response to heat shock/fever and oxidative stress signals and has been found significantly higher during bacterial infection (Borges et al., 2012; Varano Della Vergiliana et al., 2013; Tulapurkar et al., 2015). Further study is needed to investigate clinical utilization of HSPs as a potential transcriptome biomarker for early detection of recurrent or persistent infection after antibiotic therapy.

## 5 Conclusion

We found upregulated *IGHG1*, *IGHG2*, *IGHG3*, *GLV3-21*, *IGLV6-57*, *CD177*, *HIST1H2AJ*, *HIST1H2AL*, *HIST1H2BM*, *HIST1H3B*, and *HIST1H3G* genes from PBMC during active phase of *S. aureus* infection in patients with DFU at 0 weeks; these genes became downregulated at 8 weeks among healed patients, and these transcriptomes may reflect infectivity before and after antibiotic therapy. In line with the report by Sandoval-Schaefer et al. (2023) on transcriptional heterogeneity in human diabetic foot wounds, it was reported that profiling chronic foot ulcers from non-diabetic and diabetics patients using single-cell RNA sequencing display transcription changes in gene expression that may provide therapeutic baseline for treatment of DFU. Our findings further add evidence to the existing knowledge that transcriptome profiling of RNA-seq can delineate genes signatures and could be a potential valuable technique for diagnosing infection, determining severity, and measuring host immune response to therapies.

### 5.1 Clinical relevance

PBMC transcriptomes may serve as a tool for early diagnosis, monitoring and prognostication of infection, and wound healing in MSKI.

### 5.2 Limitation

This study is limited due to a small sample size, especially the group of patients who failed to heal at 8 weeks of follow-up. In addition, severity of illness, inflammatory, and immune response to infection differ from patients to patients.

## Data availability statement

The data presented in the study are deposited in the Gene Expression Omnibus (GEO) repository, accession number “GSE230426”.

## Ethics statement

The studies involving human participants were reviewed and approved by Human Research Protection and Ethics Committee (Reference No. 2000030908), Yale university. The patients/participants provided their written informed consent to participate in this study.

## Author contributions

Conceptualization and supervision: IO; Methodology: IO, JK, DZ; Validation and interpretation of results: IO, FL, TA, H-KK, JK and DZ; writing of original draft: TA; review and editing: TA and IO, funding acquisition: IO. All authors contributed to the article and approved the submitted version.
